# Instrumental variable approaches for estimating time-varying treatment effects in comparative effectiveness research

**DOI:** 10.1186/s12874-025-02625-y

**Published:** 2025-09-02

**Authors:** Daniel Tompsett, Stijn Vansteelandt, Richard Grieve, Will Dixon, Manuel Gomes

**Affiliations:** 1 Department of Primary Care and Population Health, UCL, London, UK; 2 Department of Applied Mathematics, Computer Science, and Statistics, University of Ghent, Ghent, Belgium; 3 Centre for Data and Statistical Science for Health (DASH), London School of Hygiene and Tropical Medicine, London, UK; 4 Division of Musculoskeletal and Dermatological Sciences, University of Manchester, Manchester, UK

**Keywords:** Instrumental variable, Time-varying, G-estimation, Inverse probability weighting, Rheumatoid arthritis

## Abstract

**Background:**

The increased availability of large-scale longitudinal data offers important opportunities to assess the causal effects of health interventions. In this setting, Instrumental Variable (IV) approaches have the potential to reduce the risk of bias from confounding due to unmeasured variables. However, there has been a lack of attention given to the development of IV approaches in settings when both the instrument and the potential confounders vary over time. In this paper we critically evaluate two instrumental variable approaches in time-varying settings.

**Methods:**

The paper extends an existing g-estimation method that incorporates time-fixed IVs and compares it to an inverse probability weighting approach that incorporates time-varying IVs. A simulation study investigates the relative performance of these two approaches under varying scenarios. These methods are applied to a retrospective cohort from the US National Databank for Rheumatic Diseases, evaluating the sustained use of Adalimumab (Humira) versus other biologics on the health-related quality of life of patients with Rheumatoid Arthritis. Our case study considers physicians preference for Adalimumab as an instrument.

**Results:**

The g-estimation approach provided unbiased, precise estimates of treatment effects, across a wide range of scenarios, including weak IVs and complex time-varying confounding mechanisms. The performance of the weighting approach was reasonable in scenarios with a moderate or strong time-varying IV, but deteriorated with weak IV strength. Both methods suggest that sustained treatment with Adalimumab does not improve the health-related quality of life of rheumatoid patients, compared to other biologics, but the g-estimation approach led to narrower confidence intervals.

**Conclusion:**

The proposed IV-based g-estimation approach can be reliably used in the estimation of time-varying treatments if a valid time-varying IV is available. The weighting approach offers an accessible alternative but was found to work well only when the IVs are strongly associated with treatment assignment, which is relatively unlikely in real-world applications.

## Background

### Introduction

Causal inference studies in comparative effectiveness research are increasingly time-varying in nature. Relevant data about patients and the care they receive are routinely collected over time in disease registries and electronic health records (EHRs). Such longitudinal data often provide valuable opportunities to evaluate the effect of a sustained treatment over time, e.g. the effect of taking statins for a number of years on cardiovascular disease risk. However, evaluating sustained treatment effects requires that confounders varying over time, such as trajectories of comorbidities, are controlled for.

Standard causal methods like regression adjustment, which control for all confounders at once, cannot address time-varying confounding as they block indirect effects of the treatment [1]. G-estimation is a causal inference approach from the class of g-methods [2] that were developed to handle time-varying confounding, whereby only contemporary or past confounding is controlled for at each treatment period. Standard implementation of g-estimation often makes the assumption that all sources of confounding are measured and accounted for. However, whilst this may be plausible in cases of well run data collection systems, it is often implausible in practice as important confounders (e.g. prognostic factors) are often inconsistently measured, incomplete or entirely unmeasured.

One way to address unmeasured confounding is to exploit sources of exogenous variation, instrumental variables (IVs), which are strongly associated with treatment assignment, and related to the outcome of interest only through treatment [3]. The expectation is that the IV naturally assigns individuals to different treatment groups, as-if randomised, in a way that balances both measured and unmeasured confounders. In practice, identifying IVs is challenging, with only a few sources of exogenous variation, such as genetic variants and distance to specialty care provider, being identified as potentially reliable IVs for health studies [4].

Typical IV estimation approaches, such as two stage least squares and the Wald estimator, are well established for addressing single time point interventions with time-invariant confounding [4, 5]. However in time varying settings, the cumulative effect of treatment over time, as well as trajectories of treatment effects are sought. These methods cannot afford the required flexibility to accommodate time-varying treatments [6]. Most of the seminal IV work in time-varying settings has been either theoretical [7, 8] or considered a time-varying treatment, but not time-varying IV, such as Multivariate Mendelian Randomisation studies [6, 9, 10]. Very recent work, recognising the challenges of time-varying treatments, considers machine learning or latent variable techniques to deal with time varying confounding using constructed latent IVs or related concepts [11–13], but can be complex to implement.

This paper seeks to address existing unmet challenges in implementing IV methods in time-varying settings by applying existing techniques to settings where a longitudinal variable is taken as an IV, taking its record at each follow up time as multiple instruments. This is a potentially lucrative source of multiple instruments. We seek to assess both the theoretical considerations, as well as practical challenges of such an approach. It’s unclear what estimation approaches work well across different circumstances, including varying strengths of the IV and the time-varying confounding. Secondly, there is a need to identify and assess potential sources of exogenous variation in real-world routine data sources, which can be reliably used as time-varying IV. Thirdly, there is a lack of methodological and practical guidance, and software tools, to facilitate the uptake of the methods in practice.

This paper critically evaluates and illustrates recently proposed two estimation approaches that explicitly incorporate time-varying IV to address measured and unmeasured time-varying confounding. First, we extend a g-estimation method first described in [7] and recently exploited by [6] to incorporate multiple instruments at a single time point. Second, we expand on a novel inverse probability weighting approach [14, 15], which re-weights subjects according to both treatment and IV status. We illustrate these methods in an evaluation of biologic drugs for managing patients with rheumatoid arthritis who failed to respond to non-biologic treatment. We exploit exogenous variation in physician preferences over time to instrument the assignment of different biologic treatments.

### Motivating example: US National Databank for Rheumatic Diseases

Rheumatoid Arthritis (RA) is typically treated using Disease Modifying Anti-rheumatic Drugs (DMARDs). However when patients, typically with moderate or severe symptoms, stop responding to DMARDs, then biologics, referred to as bDMARDS, are used as a second line treatment. Most clinical trials evaluate the relative effectiveness of a specific biologic compared to DMARDS [16, 17], and there is a clear lack of head to head comparisons of competing biologic drugs. Prospective studies using RA registry data can provide valuable evidence on the effectiveness of alternative biologics.

Our example uses data from the US National Databank for Rheumatic Diseases (FORWARD) [18] to evaluate the relative effectiveness of Adalimumab over other biologics. FORWARD collects longitudinal data from over 50,000 patients, across 1,500 rheumatologists in the US and Canada. It includes rich information about RA patients and the care they receive, collected biannually through either email or post-based patient-reported questionnaires.

#### Target population, treatment strategies and outcome

Our subset of FORWARD contained data on 17921 participants in the US diagnosed with moderate and severe RA, spanning from the 1 st January 2005 to 30th June 2020. Patient data was recorded at 6 monthly intervals labeled as ‘phases’, collected by a series of bi-annual questionnaires which record relevant patient data for the preceding 6 month period. We considered $T=4$ phases, with Phase 1 corresponding to the baseline period, the first phase biologic treatment initiation was recorded. Follow up data was collected at phases 2, 3 and 4, with the end-of-study outcome taken at phase 4, 18 months after initiation.

Our target population were patients who failed to respond to conventional DMARDs and initiated a biologic drug. Patients were included in the sample if they had data for at least 3 follow-up phases and remained on bDMARDs for that period. A small proportion (less than 10%) of individuals who initiated biologics but switched back to conventional DMARDS at some point during follow up were excluded, which may have induced some level of selection bias. We also had to exclude patients for whom physician information (source of exogenous variation used to specify the instrument - see IV-based G-estimation (IV-G) )was not available. The final sample included 1952 patients.

The treatment strategies were defined as ‘initiating and taking Adalimumab for 18 months’ versus ‘initiating and taking any other biologic over that period’. The estimand of interest was the (total) average treatment effect (ATE) of taking Adalimumab compared to other bDMARDs on the health outcome at 18 months. The outcome of interest was the quality-adjusted life year (QALY), estimated using the EuroQOL-5D (EQ-5D) health-related quality of life measure. EQ-5D is a continuous measure, anchored a scale where 0 indicated death and 1 indicates perfect health. These are derived from a 3 point scale prior to 2011 (EQ5D-3L) or 5 point scale (EQ5D-5L) from 2011 onwards, on 5 areas of life quality. The QALY over the 18 month follow up, was defined from EQ-5D scores as follows

$$\begin{aligned} \text {QALY} & =0.25*\text {EQD}_1+0.5*\text {EQ5D}_2+0.5*\text {EQ5D}_3\\ & \quad +0.25*\text {EQ5D}_4 \end{aligned}$$

where $\text {EQ5D}_1$ is a patient’s EQ-5D score at time 1. Thus the QALY ranged from 0 to 1.5, and captured a weighted indicator of how many years of good quality of life one can expect over that period. For example, a QALY of 1.5 would indicate a full 18 months in full health.

Baseline characteristics are reported in Table 1. This includes (time-invariant) baseline characteristics such as age, gender, race and smoking status, as well as time-varying characteristics, including health insurance, duration of RA symptoms, disease activity (DAS), health assessment questionnaire (HAQ) and other comorbidities. We also considered other medications, and whether patients were recorded to have taken more than one bDMARD during one phase. At baseline, patients who initiated Adalimumab (*n*=648) were younger and healthier than those initiating another bDMARD (*n*=1304). For example, the latter were associated with a higher average comorbidity score and years of symptoms.

**Table 1:** Baseline characteristics at time $t=1$ for the final sample of RA patients

Variable	Non-Adalimumab bDMARDs (*n*=1304)	Adalimumab (*n*=648)	*p*
Age (SD)	60.5 (11.3)	58.7 (10.9)	<0.01
Male (%)	17.9	20.7	0.14
White (%)	93.6	92.0	0.21
Currently Smoke (%)	55.4	61.4	0.06
Medicare (%)	38.0	27.6	<0.01
Duration of RA Symptoms in Years (SD)	16.3 (12.3)	15.1 (11.4)	0.03
0-9 Comorbidity Index	1.82	1.64	0.016
Disease Activity Score $>3.2$ (%)	41.2	40.6	0.78
0-10 Pain Scale (SD)	3.55 (2.64)	3.48 (2.67)	0.56
EQ-5D Score (SD)	0.75 (0.17)	0.76 (0.17)	0.16
HAQ Disability Score (SD)	1.06 (0.72)	1.01 (0.69)	0.16
Heart Disorder (%)	7.2	6.4	0.54
Pulmonary Disorder (%)	7.4	4.6	0.01
Diabetic (%)	10.9	8.6	0.11
Infection (%)	45.2	42.7	0.29
Cancer (%)	2.2	3.1	0.24
Current csDMARD use (%)	76.1	78.5	0.22
Current Opioid use (%)	30.6	25.9	0.025
Total no. of DMARD prescriptions	2.26	2.21	0.45
$>1$ prescribed bDMARD this phase (%)	8.8	13.6	<0.01

#### Time-varying IV

We exploited exogenous variation in physician’s preferences for Adalimumab over time as an IV for treatment assignment. Prescribing preferences have been widely used as IV for evaluating point treatments [19–21], and we investigated its plausibility in a time-varying setting. We considered three definitions for the time-varying IV. Firstly, we took the proportion of Adalimumab prescriptions (compared to all biologic prescriptions) by each doctor over the 6 month period. In this case, the instrument took the value 1 if the within-physician proportion of Adalimumab prescriptions was above the median of the average proportion for all physicians in that calendar phase, and 0 otherwise. The second definition involved setting the IV to 1 if Adalimumab was the physician’s most prescribed biologic, 0 otherwise. Lastly, we defined the IV according to physician’s last issued biologic prescription, with the IV being set to 1 if it was Adalimumab, 0 otherwise.

## Methods

### General time-varying data setup

**Fig. 1:**
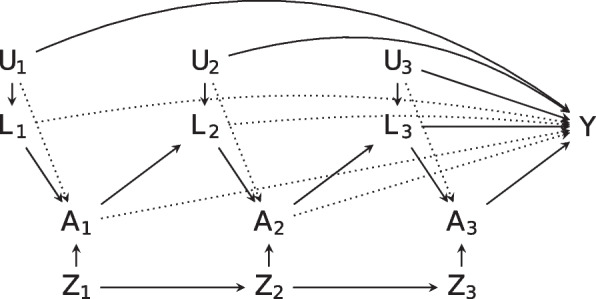
DAG displaying setup of time-varying data for $T=4$. The effect of time-varying treatment at time *t*, $A_t$, on outcome *Y* is the direct association between $A_t$ and *Y*, and the associations mediated through future time-varying measures $L_{t+1}$. For example $A_2 \rightarrow L_3 \rightarrow Y$. This effect is confounded by observed measures $L_t$, as well as unmeasured time varying measures $U_t$. At each *t* a time varying instrument $Z_t$ is recorded, directly associated with $A_t$ but not *Y*. Note that dashed lines are used for visual clarity and serve the same intent as solid lines

We followed closely the time-varying data setup adopted by ‘Micheal et al’ [14], shown in Fig. 1. Suppose we have *n* patients over *T* time periods (for illustration we set $T=4$), for which we observe a treatment $A_t$, $t=1,\ldots,t$, up to time $T-1$. For the rest of the paper we will refer to ‘treated’ $(A_t=1)$ and ‘untreated’ $(A_t=0)$ groups. We observe a continuous end-of-study outcome *Y* at time *T*, and both observed and unobserved time-varying confounders $L_t$ and $U_t$, respectively. $A_t$ affects *Y* directly and indirectly via $L_t$ and $U_t$. We define $Z_t$ as the time-varying instrument at time *t*. A more complex setup is shown in the Appendix and considered in the simulation study.

We define $A=(A_1,A_2,A_3)$, $Z=(Z_1,Z_2,Z_3)$, $L=(L_1,L_2,L_3)$ and $U=(U_1,U_2,U_3)$. Let $\bar{A}_t$, be the vector holding the history of *A* up to time *t*. Subject specific values are defined by subscript *i*, such as $A_{1i}$. When a variable is referred to in bold, this represents the matrix of values of that variable for each of the *n* individuals, for example $\textbf{A}_1=(A_{11},\ldots,A_{1n})'$. We define *Y*(*a*) as the counterfactual outcome of *Y*, if an individual treatment history were set to some treatment set $a=(a_1,a_2,a_3)$. A causal effect between *A* and *Y* is sought via estimating the Average Treatment Effect (ATE), the average difference in the counterfactual outcome in the population under two relevant treatment regimes.

$$\begin{aligned} E[Y(1,1,1)-Y(0,0,0)]. \end{aligned}$$

### IV assumptions

Intuitively an IV *Z* can be thought of as a variable that is associated with treatment and for which any association with an outcome *Y* only occurs through its association with treatment *A*. By estimating the $Z-A$ association, we can identify the $A-Y$ association despite confounding by *L* or *U*. The simplest demonstration of this idea in time fixed settings is the Wald estimator, where the $Z-Y$ association is divided by the $Z-A$ association, leaving behind the $A-Y$ association, provided this association is linear [2].

The main IV assumptions involve multivariate extensions of the three main IV assumptions in time-fixed settings. We assume that positivity and counterfactual consistency hold for *Y*(*a*) at all time points [2], and that *L* and *U* represent all possible confounding between *Y* and *A* outside of treatment history.

1. IV1 (relevance): requires that there is a measurement of *Z* and *A* at each time point, i.e. as many instruments as treatment assignment points, and there exists a suitably strong association between $Z_t$ and $A_t$ at each time point. If this association is not strong enough, estimates may suffer from weak instrument bias [22]. For further details see Appendix.
2. IV2 (Conditional Exchangeability): $Z_t$ does not share any common causes with *Y*, conditional on the set of variables $M_t$. In this paper we set $M_t=(\bar{A}_{t-1},\bar{Z}_{t-1})$, that is the history of treatment and instrument, which we consider the typical minimal set for $M_t$. The set $M_t$ may also include some subset of $\bar{L}_t$. The conditional exchangeability assumption essentially expresses that $Z_t$ should not depend on unmeasured common causes $U_t$ of treatment and outcome.
3. IV3 (Exclusion Restriction): $Z_t$ cannot have any direct effect on *Y* other than through *A*, nor does it affect future measured or unmeasured confounding ($Z_t$ may affect future values of *Z* or *A*).

IV2 and IV3 are often formalised by the single assumption $Y(a) \perp \!\!\!\perp Z_t| M_t\; \text {for}\; t \; and\; a$. The following methods make additional assumptions about the nature of the data, which is noted in the relevant sections describing the methodologies.

In practice, only IV1 is testable from the available data. Assessment of IV1 is based on determining the strength of association between $Z_t$ and $A_t$. In some cases the $A_t-Z_t$ correlation is used to determine IV strength. However more formal tests have been developed, notably the Stock Yogo F-test [23] and other related measures. However most of these tests are only available in point treatment settings. Recently an F-statistic has been developed for settings with time varying treatments and multiple IVs [10].

Although IV2 and IV3 are not testable from the data, it is common to check the balance of measured confounders between IV groups as an indicator of exhangeability, though imbalances in unmeasured confounders cannot be determined. Otherwise, expert opinion, sensible definitions of the IV, logical argument, or prior analyses can be used to justify if an IV is not associated with confounders or directly impacting outcome.

### IV-based G-estimation (IV-G)

#### Overview

Our proposed g-estimation approach builds on a recent proposal that incorporates multiple instruments within a single time period [6, 7]. We seek to fit the parameters $\beta _t$ of a Structural Nested Mean Model (SNMM):

1 $$\begin{aligned} & E[Y(\bar{a}_t,0)-Y(\bar{a}_{t-1},0)|\bar{A}_t=\bar{a}_t, \bar{Z}_t, M_t]=\beta _t a_t \quad t=1,\ldots,T-1. \end{aligned}$$

where $Y(\bar{a}_t,0)$ is the counterfactual outcome under a patient’s observed treatment history up to time *t*, and untreated afterwards. Specifically, $\beta _t$ models $E[Y(a_t,0)-Y(a_{t-1},0)|\bar{A}_t=\bar{a}_t, \bar{Z}_t, M_t]$, the shift in the counterfactual mean outcome if someone was treated at time *t* (versus not treated).

We require an assumption that the shift in conditional counterfactual outcome is the same in the treated $A_t=1$ as the untreated $A_t=0$ groups:

$$\begin{aligned} & E[Y(\bar{a}_t,0)\!-\!Y(\bar{a}_{t-1},0)\!|\!\bar{A}_{t-1}\!=\!\bar{a}_{t-1}, A_t=1,\bar{Z}_t,M_t]\\ =&E[Y(\bar{a}_t,0)\!-\!Y(\bar{a}_{t-1},0)\!|\!\bar{A}_{t-1}\!=\!\bar{a}_{t-1}, A_t=0,\bar{Z}_t,M_t]. \end{aligned}$$

This is a multivariate extension of the no current treatment interaction assumption [3] which holds if the true relationship between the $A_t$ and *Y* is linear with no interaction effects. It can be shown that, under the no current treatment interaction assumption, we can derive the ATE as the sum of the parameters $\beta _t$ which we demonstrate in the Appendix. In [6] it was assumed that all instruments were measured at baseline and were independent of prior variables or each other. Here we extend this method to incorporate a time-varying instrument as described in [7].

In essence g-estimation aims to identify the $\beta _t$ that results in the independence of the counterfactual outcome under no treatment, *Y*(0, 0, 0) and $Z_t$ (given $M_t$), which we have assumed is true when assumptions IV2 and IV3 are met. For example if *Z* was the randomisation indicator, this would be expected to be independent of outcome under any treatment regime. By removing the effect of treatment of outcome as defined in Eq. (2), to estimate *Y*(0, 0, 0), we could then set $\beta _t$ to that which ensures the expected scenario that randomisation is independent of outcome.

#### Method

Formally, IV-G is performed as follows. The SNMM, if correctly specified, allows us to define an estimate of the counterfactual under no treatment *Y*(0, 0, 0) as

$$\begin{aligned} Y(0,0,0)=H_1=Y-\sum _{t=1}^T\beta _t A_t. \end{aligned}$$

IV2 and IV3 then imply that

$$\begin{aligned} H_1 \perp \!\!\!\perp Z_t| M_t\; \forall a\qquad t=1,\ldots T. \end{aligned}$$

IV-G seeks to find the $\beta _t$ such that, in a regression model of $H_1$ and $M_t$ on $Z_t$, the coefficient of $H_1$ would be zero for all *t*, thus satisfying IV2 and IV3. It can be shown [2, 6] that the $\beta _t$ that satisfy these conditions can be found by solving the following estimating equations

$$\begin{aligned} \sum _{i=1}^n (H_{1i})(Z_{ti}-E[Z_{ti}|M_{ti}])=0\qquad t=1,2,3 \end{aligned}$$

with subscript *i* denoting the subject specific values for *H*, *Z* and *A*, and values for $E[Z_{ti}|M_{ti}]$ estimated via a logistic regression model of $P(Z_t=1|M_t)$. These equations have a closed form solution:

$$\begin{aligned} \hat{\beta }=\textbf{Y}^T(\textbf{Z}-\mathbf {E(Z)})(\textbf{A}^T(\mathbf {Z-E(Z)}))^{-1} \end{aligned}$$

We can obtain standard errors and confidence intervals for $\beta$ using non-parametric bootstrap.

### Inverse probability of IV weighting (IV-W)

#### Overview

Our second approach, IV-W [14, 24], is an inverse probability weighting method which seeks to fit the parameters of a Marginal Structural Model (MSM).

2 $$\begin{aligned} E[Y(a)]= \beta _0+\sum _{t=1}^T \beta _t a_t. \end{aligned}$$

which directly models the counterfactual outcome under a specific regime *a*. Under the no current treatment interaction assumption, we show (see Appendix) that the SNMM of Eq. (1), implies MSM of Eq. (2), ensuring a fair comparison between methods. The estimand of interest is the effect of always treating versus never treating in the population of interest, i.e. $E[Y(1,1,1)-Y(0,0,0)]$. For the MSM, this estimate directly targets the ATE, which corresponds to the sum of the $\beta _t$, as with the SNMM.

IV-W is an IV analogue to conventional Inverse Probability Weighting. Conventional inverse weighting creates a pseudo-population that balances measured confounders within the treated and untreated groups, by directly modeling the relationship between the treatment and confounders. IV-W takes advantage of the fact that a relevant IV affects a patients assignment to treatment in a way that is ‘balanced’, or unaffected by confounding. The simplest example is if *Z* is randomisation to treatment. Though actual treatment taken may depend on socio-economic or health characteristics, randomisation strongly affects taken treatment in a manner not impacted by any of these variables.

IV-W uses this balanced treatment assignment process defined by the IV as a means to construct weights that create a balanced pseudo-population for the treatment that does not need to control for confounders.

#### Method

The IV-W approach first defines the weights $\bar{W}^{IV}$ as a function of a binary treatment $A_t$ and binary instrument *Z*.

$$\begin{aligned} \bar{W}^{IV}=\prod _{t=1}^T W_t^{IV} \end{aligned}$$

$$\begin{aligned} W_t^{IV}=(-1)^{1-Z_t}P_{Z_t}(Z_t|M_t)\Delta _t \end{aligned}$$

where $$P_{Z_t}(Z_t|M_t)=Z_tP(Z_t=1|M_t)+(1-Z_t)$$$$P(Z_t=0|M_t)$$ denotes the probability mass function of $$Z_t$$ given $$M_t$$. We define $$\Delta _t$$ being the difference in the probability mass function $$P_{A_t}$$ for treatment $$A_t$$ given $$Z_t$$, $$L_t$$ and $$M_t$$, when $$Z_t$$ is set 1 versus set to 0

$$\begin{aligned} \Delta _t\!=\!P_{A_t}(A_t|Z_t\!=\!1,M_t,\bar{L}_t)\!-\!P_{A_t}(A_t\!|\!Z_t\!=\!0,M_t,\bar{L}_t) \end{aligned}$$

where $$P_{A_t}(A_t|Z_t,M_t,\bar{L}_t)=A_tP(A_t=1|Z_t,M_t,\bar{L}_t)+$$$$(1-A_t)P(A_t=0|Z_t,M_t,\bar{L}_t)$$. We note that $$\Delta _t$$ can depend on variables within $$M_t$$ or measured confounders such as $$\bar{L}_t$$ [14]. It can be shown [14] that subsequently, under additional assumptions given later, consistent estimates of $$\beta _t$$ can be attained from the following estimating equations

$$\begin{aligned} \sum _{i=1}^n h(A_i) \frac{Y_i-(\beta _0+\sum _{t=1}^{T-1}\beta _t A_i)}{\bar{W_i}^{IV}}=0 \end{aligned}$$

Here $h(A_i)$ is a user chosen function of $A_i$ of the same dimension as $\beta$, the obvious choice being $h(A_i)=(1,A_i)=A_i^{'}$, the vector $A_i$ with a first column containing 1. When we take $h(A)=A^{'}$ these estimating equations have the closed form solution of a weighted least squares estimator

$$\begin{aligned} \hat{\beta }=\left( \frac{\mathbf {A^{'T}A^{'}}}{\textbf{W}^{IV}}\right) ^{-1}\frac{\mathbf {A^{'T}Y}}{\textbf{W}^{IV}}. \end{aligned}$$

with $\textbf{W}^{IV}$ a vector containing the weights $\bar{W}_i^{IV}$ for each of the *n* patients. As with g-estimation, standards errors and confidence intervals can be obtained via bootstrap [14].

This method requires further assumptions. Firstly the method is currently only designed for cases where $Z_t$ and $A_t$ is binary. We also require the ‘independence compliance type’ assumption [14, 15], where (if $Z_t$ was randomisation to treatment), sets a specific orthogonality condition on the compliance with randomised treatment.

$$\begin{aligned} & P_{A_t}(A_t|Z_t=1, M_t,\bar{L}_t,\bar{U}_{t})\\ - & P_{A_t}(A_t|Z_t=0,M_t,\bar{L}_t,\bar{U}_{t})=\Delta _t \end{aligned}$$

This assumption is necessary for calculation of $\Delta _t$ as it ensures it does not depend on unmeasured confounding. This is used instead of the ‘no current treatment interaction’ assumption, necessary for IV-G.

The weights $\textbf{W}^{IV}$ can be estimated using generalised linear models of $Z_t$ on $M_t$, and $A_t$ on $M_t$, $L_t$ and $Z_t$ [15], or via a specific Maximum Likelihood Estimation (MLE) approach used in [14]. These are not weights in the traditional sense, as they are centred around zero and can take negative values. If $Z_t$ was randomisation, then those who always take the treatment which they are randomised to will be given a positive weight, and vice versa. An established issue is that their stability is heavily dependent on the magnitude of the $\Delta _t$ term, which can quickly approach zero when the association with $Z_t$ and $A_t$ is not strong, and create unstable weights.

To help deal with unstable weights, established work [14, 15] proposed dividing $W_t^{IV}$ by the term $St_t^{Stab}=P_{A_t}(A_t|\bar{A}_{t-1})$, denoted hereafter as ’standard’ stabilised weights.

This paper proposes an alternative ’Delta’ stabilised weights, which divides the $W_t^{IV}$ by the term

$$\begin{aligned} \Delta _t^{Stab}\!=\!P_{A_t}(A_t|Z_t\!=\!1,\bar{A}_{t-1})\!-\!P_{A_t}(A_t|Z_t\!=\!0,\bar{A}_{t-1}) \end{aligned}$$

that is a term similar to $\Delta _t$ with dependence on any $L_t$ and seeks to stabilise small terms for $\Delta _t$.

### Simulation study

The aim of the simulation study is to investigate the performance of IV-G and IV-W across a broad range of settings. We first consider more ideal circumstances, with large sample sizes, ’strong’ time-varying IVs (i.e. highly predictive of treatment assignment) and relatively simple associations between variables. We then consider more realistic circumstances informed by the case study, including weaker IVs, low sample sizes and a more complex time-varying data setup.

We simulate data according to the DAG in Fig. 1. This is the simulation setup that was considered in prior works on IV-W [14], which we follow as to allow for fair comparison both with IV-G methods and previous results. A more complex DAG, with variables at each time more directly affected by their history and other past variables is considered in the Appendix. Our data generating mechanism (DGM) is as follows.

- The time-varying instrument $Z_t$ is simulated as
  $$\begin{aligned} logit(P(Z_t=1|Z_{t-1},A_{t-1}))=&\sigma _{0t}+\sigma _{1t}Z_{t-1}+\sigma _{2t}A_{t-1} \end{aligned}$$
  with $$\sigma _{0t}=0$$, $$\sigma _{1t}=0.5$$ and $$\sigma _{2t}=0$$.
- $L_t=\lambda _{0t}+\lambda _{1t}A_{t-1}+\lambda _{2t}U_t$. We set $\lambda _{0t}=\lambda _{1t}=1$ and $\lambda _{2t}=0.33$ for all *t*.
- $A_t$ is generated using
  $$\begin{aligned} & P(A_t=1|A_{t-1},Z_t,Z_{t-1},L_t,U_t)\\ =&\Phi (\mu _0+\mu _1 L_t+\mu _2 U_t+\mu _3 A_{t-1}+\mu _4 Z_{t-1})(1-\Delta _t)+Z_t\Delta _t. \end{aligned}$$
  with $\Phi$ denoting the standard normal cdf and $\mu =(\mu _0,\mu _1,\mu _2,\mu _3,\mu _4)=(-0.2,0.2,0.2,0,0)$.
- Lastly, we construct the outcome by computing
  $$\begin{aligned} Y=\mathbf {\beta }_U \textbf{U}+ \mathbf {\beta }_L \textbf{L}+\beta _{t} \textbf{A}+\epsilon \end{aligned}$$
  where $\beta _{U}=(1/3,1/3,1/3)$, $\beta _L=(1,1,1)$, $\beta _A=(1,1,1)$ and $\epsilon$ follows a standard normal distribution.
- We simulate baseline variables first $(t=1)$, then generated subsequent time periods in ascending order.
- For simplicity, $\Delta _t$ is assumed to be a constant between 0 and 1 (with no dependence on *A* or *Z*), which closely approximated the correlation strength between $Z_t$ and $A_t$.
  $$\begin{aligned} \Delta _{t}=\Phi (\alpha _t). \end{aligned}$$

The true values for $\beta _t$ are (2, 2, 1), and the estimand of interest was the ATE with a true value of 5.

#### Implementation

All simulations, and the case study analysis are performed using the software package ‘R’ [25] and based mostly on user-written functions. The R code for implementing IV-G and IV-W is available as supplementary material. We generated $m=1000$ datasets, with each dataset being bootstrapped $b=1000$ times. The methods IV-G and IV-W were implemented as described in Methods, with $P(A_t=1|Z_t,M_t,L_t)$ and $P(Z_t=1|M_t)$ modeled correctly using logistic regression, and used to create estimates for $\Delta _t$. The simulations considered both standard and delta stabilising weights defined as IV-W-St and IV-W-D, respectively. In cases of unstable results, we removed the results of simulations with extreme values of the ATE, defined as the upper or lower quartile of the estimates of the ATE plus or minus one and a half times the IQR.

We computed the ATE by summing up the $\beta _t$ estimates. For each dataset, we took the average value of the ATE across the 1000 bootstraps, and computed the 95% confidence intervals (CI) using the percentile method, which takes the 2.5th and 97.5th percentiles of the ordered bootstrap estimates.

We varied two key parameters in the simulation: 1) alternative sample sizes *n* of 1000, 5000 and 10000; 2) the parameters $\alpha _t$. Specifically the simulation is designed so that $\Delta _t$ is approximately the $Z_t-A_t$ correlation coefficient. We adopted commonly used definitions of correlation levels [26] and set coefficients below 0.1 as low to negligible strength, and above 0.7 to be very strong. We set $\alpha _t$ at each time *t* to either $-1.2,-0.5,0$ or 0.5, which corresponded to ‘weak’ (0.1), ‘moderate’ (0.3), ‘strong’ (0.5) or ‘very strong’ (0.7) values for $\Delta _t$.

#### Performance measures

We reported the average bias of the ATE, computed as difference between the average over the *m* datasets and its true value. We reported the Root Mean Square Error (RMSE) to combines both bias and efficiency, and Monte Carlo simulation error (MCE) as defined in [27]. We also reported CI coverage, computed as the proportion of simulations for which the CI interval holds the true ATE value of 5.

To further assess IV strength we computed the Sanderson-Windmeijer conditional F-statistic $(F_{SW})$ [10]. An $F_{SW}$ statistic is calculated for each $A_t$ indicating the ability of *Z* to predict $A_t$ after *Z* has already been used to predict the remaining $A_t$. Sanderson and colleagues [10] suggested that if the $F_{SW}$ is above 10 for each $A_t$, *Z* is considered sufficiently jointly predictive of all $A_t$, though recent work in the context of time-fixed IVs suggested that much higher $F_{SW}$ values may be required for IV to perform well [28].

### Case study: comparative effectiveness of Adalimumab for rheumatoid arthritis

We applied the methods to a comparative assessment of Adalimumab versus other biologics for the management of rheumatoid arthritis in patients who failed to respond to non-biologic drugs. IV1 is likely to hold if the IV is defined according to the proportion of prescriptions and the most prescribed biologic. Around 10 percent of patients showed a change in IV status at each treatment period, with conditional F-statistics ranging between 50 and 80. We decide to use dichotomised prescription proportion, the strongest IV, for the analysis. We did not consider the instrument based on the last biologic prescription because it showed very weak conditional F-statistics (around 6).

Preference at initiation (See Appendix) remains weakly influenced by having medicare, being diabetic, history of DMARD use and comorbidity index, but not strongly by other health related measures. Adalimumab is an expensive treatment covered by Medicare, and is known to impact blood glucose levels. Hence it is not implausible that a prescriber’s belief in Adalimumab as a preferential treatment depends partly on the certain trajectories of health they observe in their patients. As our end of study outcome is a measure derived from EQ-5D, there is a risk that preference may depend on history of these scores. The validity of assumption IV2, may be strengthened if we consider an analysis that adjusts for these confounders. We feel that any other effect of preference on EQ-5D would be through the resulting treatment they are assigned, and thus believe that IV3 is likely to hold.

We fitted two main effects logistic regression models for $Z_t$ to estimate $\Delta _{t}$ and $E[Z_t|M_t]$. The first model included the history of treatment, EQ-5D score and physician’s preference (IV). The second model included further adjustment for the additional confounders above, which we denote ‘adjusted’. We used $b=1000$ bootstrapped datasets to obtain percentile confidence intervals.

IV-W was performed using the ‘standard’ stabilised weights, given that the delta approach showed little improvement in the simulations. To combat the extreme weights identified in the simulations, we consider a trimming approach [29]. Weights above or below a certain threshold are set to those threshold values. We consider the upper quartile plus 1.5 times the Inter-Quartile Range (of the weights) and the lower quartile minus 1.5 times the Inter-Quartile Range as these thresholds. This is a stronger threshold than typical (normally the 1 st and 99th percentiles), necessary to help control the weights.

## Results

### Simulation study

**Fig. 2:**
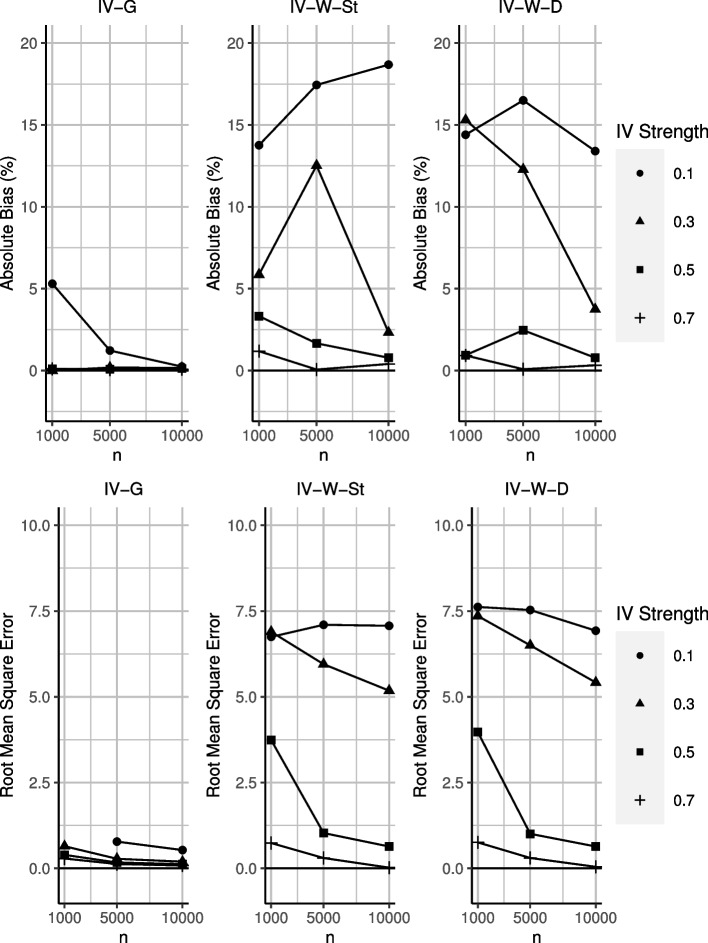
Absolute bias and root mean square error from IV-G and IV-W. IV-G result for the scenario with weak IV and *n*=1,000 is not shown as it exceeded the upper bound of the y-axis

**Fig. 3:**
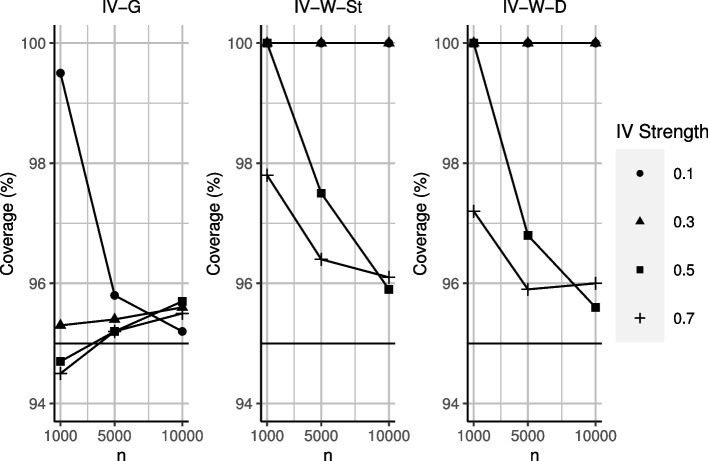
CI coverage results from IV-G and IV-W. IV-W results for scenarios with weak IV are not shown as they exceeded the upper bound of the y-axis. Nominal coverage is 95%

The main results are shown in Figs. 2 and 3, and Tables 2 and 3. IV-G reported unbiased estimates and CI coverage close to nominal levels across most scenarios. The performance of this approach began to deteriorate in the scenario that combines weak IV strength and low sample size. We reported the average and minimum conditional F-statistic in the (Appendix), which suggested that the performance of IV-G deteriorates specifically when the F-statistic falls below the threshold of 10.

**Table 2:** Simulation results for IV-G, based on b=1000 bootstrapped samples

*n*	*Cor*(*Z*)	Bias	RMSE	MCE	Coverage
10000	0.7	0.004	0.008	0.003	95.5
10000	0.5	0.006	0.122	0.004	95.7
10000	0.3	0.008	0.195	0.006	95.6
10000	0.1	0.012	0.533	0.016	95.2
5000	0.7	−0.003	0.127	0.004	95.2
5000	0.5	−0.004	0.170	0.005	95.2
5000	0.3	−0.009	0.279	0.009	95.4
5000	0.1	−0.061	0.780	0.025	95.8
1000	0.7	0.005	0.286	0.009	94.5
1000	0.5	0.005	0.394	0.012	94.7
1000	0.3	−0.000	0.646	0.020	95.3
1000	0.1	0.265	21.633	0.684	99.5

**Table 3:** Simulation results for IV-W, based on b=1000 bootstrapped samples

Weight	*n*	*Cor*(*Z*)	Bias	RMSE	MCE	Coverage
St	10000	0.7	−0.020	0.021	0.007	94.7
St	10000	0.5	−0.039	0.637	0.020	96.4
St	10000	0.3	0.116	5.180	0.180	100.0
St	10000	0.1	0.934	7.071	0.239	100.0
Delta	10000	0.7	−0.016	0.045	0.007	96.0
Delta	10000	0.5	−0.039	0.637	0.020	95.6
Delta	10000	0.3	0.187	5.417	0.186	100.0
Delta	10000	0.1	0.670	6.927	0.237	100.0
St	5000	0.7	−0.003	0.302	0.010	95.1
St	5000	0.5	−0.083	1.030	0.033	97.2
St	5000	0.3	0.626	5.951	0.205	100.0
St	5000	0.1	0.872	7.100	0.242	100.0
Delta	5000	0.7	−0.004	0.303	0.010	96.1
Delta	5000	0.5	−0.123	1.007	0.032	96.8
Delta	5000	0.3	0.614	6.501	0.222	100.0
Delta	5000	0.1	0.825	7.531	0.256	100.0
St	1000	0.7	−0.059	0.738	0.024	96.6
St	1000	0.5	0.166	3.740	0.128	99.9
St	1000	0.3	0.293	6.896	0.236	100.0
St	1000	0.1	0.688	6.747	0.232	100.0
Delta	1000	0.7	−0.046	0.757	0.024	96.1
Delta	1000	0.5	0.047	3.973	0.138	100.0
Delta	1000	0.3	0.765	7.357	0.252	100.0
Delta	1000	0.1	0.720	7.620	0.260	100.0

In scenarios with a strong IV strength (correlations of 0.7 and 0.5) and moderate to large sample sizes, IV-W provided unbiased results and CI coverage somewhat above nominal levels. This approach, as is generally expected of inverse weighted procedures, performed worse in terms of RMSE compared to IV-G.

For weaker IVs (correlations of 0.3 and 0.1), IV-W led to more pronounced biases and overcoverage, producing 100% coverage irrespective of the sample size. This combination of both significant bias and overcoverage indicates high instability in the estimated ATE between each simulated dataset. In fact in some cases (up to 10%), the procedure could not converge to a result. This behaviour is indicative of either extreme weights that blow up toward infinity, or that, at least for IV-W, the IVs were no longer sufficiently strong. The performance of IV-W did not differ according to the approach (standard and delta) taken for stabilising the weights.

The Appendix reported the results for the same scenarios.

### Case study

ATE and 95% CI for Adalimumab versus other biologics initiators are reported in Table 4. IV-G suggested a small improvement in QALY at 18 months for patients initiating Adalimumab vs other biologics. However, IV results according to any approach did not reject the null of no difference in effect (all confidence intervals crossed zero). Adjusting for confounding variables potentially related to preference did not meaningfully change the results.

**Table 4:** ATE and 95% CI for the effect of Adalimumab vs other biologics on the 18 month QALY

Method	ATE	95% Confidence Interval
IV-G	0.028	(−0.003, 0.059)
IV-G (Adjusted)	0.022	(−0.002,0.044)
IV-W	−0.138	(−0.641, 0.186)
IV-W (Adjusted)	−0.074	(−0.56, 0.186)
IV-W Trimmed	−0.041	(−0.157, 0.085)
IV-W Trimmed (Adjusted)	−0.024	(−0.158, 0.078)

In IV-W Trimmed, weights are capped to the upper quartile plus 1.5 times the IQR, or the lower quartile minus 1.5 of the distribution of the weights

Without any trimming of extreme weights, IV-W results produced very wide 95% CIs and large effects. This indicates the method potentially struggling with the same issue as was found in simulation studies. After trimming, IV-W attained estimates more in line with those of IV-G, though CIs remain wide, and results may have been biased due to insufficient control for confounding as a result of the trimming. Similar results were found when the ‘delta’ stabilised weights were used instead.

## Discussion

### Contribution

This study adds to recent efforts to use IV analysis in comparative effectiveness research with time-varying treatments and confounding. We applied the g-estimation methodology to incorporate time-varying instruments, and contrasted it against a weighting approach. IV-G is shown to be unbiased, with nominal coverage across a wide range of realistic scenarios with simple and complex time-varying confounding mechanisms. The performance of IV-W was reasonable in scenarios with a strong time-varying IV, but quickly deteriorated with weak IV strength. We illustrated the practical challenges of implementing time-varying IVs in a comparative effectiveness study of biologics for rheumatoid arthritis, exploiting exogenous variation in prescribing preferences over time. IV-G and IV-W led to similar results about the effect of initiating Adalimumab versus other biologics, but the latter approach led to wider confidence intervals.

This paper makes several contributions to the literature concerned with the use of time-varying IVs. Firstly, we provided a head-to-head comparison of the g-estimation approach with a recently proposed weighting approach [14]. On the one hand, applied users are often more familiar with weighting than g-estimation procedures so the former tends to be more readily accessible. On the other hand, weighting approaches are known for being more uncertain due to unstable weights, and hence it was of interest to compare its relative performance against g-estimation. For settings where the time-varying IV is strongly associated with treatment assignment, our findings that the IV-W perform well are very much in line with those of ‘Micheal et al’ [14].

Unlike previous work, our study also explored settings with weak and moderate IV strength, and found that the IV-W is not recommended in these circumstances. None of the proposed stabilised weights helped mitigate the issue with extreme weights, with the extent of the biases being a function of the magnitude of the delta term. This method was also hindered by requiring a binary treatment and IV.

Secondly, we built on recent developments on Mendelian Randomisation analysis of time-varying treatments, and proposed a g-estimation approach that explicitly incorporates time-varying instruments. Using a conditional F-statistic, designed for the Mendelian Randomisation setting, we found that the IV-G approach is likely to perform well in practice for instruments which strength is above the relatively well-established threshold of 10. While g-estimation of SNMM does not directly target the population ATE, we showed that the causal parameters can be interpreted as ATE under the additional no current treatment interaction assumption.

Thirdly, as part of the case-study, we explored whether exogenous variation associated with prescribing preferences could be potentially used as a time-varying IV. This is valuable because: i) there are fewer sources for time-varying compared to time-fixed instruments, e.g. genes do not vary over time; ii) certain sources of IVs might not be available in routinely collected data, such as the distance to speciality care provider, which was considered in recent studies [14, 15]. Our case-study suggested that physician preferences for Adalimumab were moderately associated with the choice of biologic treatment for the management of rheumatoid arthritis, but their strength decreases over time.

### Limitations, guidance and further research

The methods of the paper share the limitation of time fixed IV methods in that violations of the IV assumptions can lead to significant bias. We test violations of IV1 via simulation study, but the time varying nature of the IV may also increase the risk of IV dependence on time-varying confounding and violating IV2 or IV3. The case study highlights practical challenges regarding IV strength. A time-varying IV in real world data may lose its strength quickly, with the IV at baseline accounting for much of the association with treatment over time. Information about physicians was missing for 60% of the individuals, and with only complete cases analysed. This both led to issues with selection bias and had direct implications for the strength of the IV at later times and length of the follow up. Missing data could be imputed in practice, however constructing an IV from imputed data has little precedent and is not recommended. The best means to mitigate this issue is to have good data collection practices to minimise missing data.

In practice, users of either method, should carefully check the strength of the IV after baseline using an appropriate F-test and consider whether past history is likely to directly affect how the IV develops over time. If using the IV-W methodology, weights should be carefully assessed for large anomalous values, or for estimated values of $\Delta _t$ close to zero, as a clue to unstable weights. In the case study, an ad-hoc trimming of the weights [30] was used, and may help in scenarios with only a few extreme weights or IV strength that is borderline, but is largely ineffective in cases of weak instruments.

Another question is whether to construct an IV using use only recent prescriptions or the whole prescription history. We recommend, as in the case study, to compute the instrument based on the whole relevant history (in our case all physician’s prescriptions) up to that time, but it could have instead considered history only in that time period.

Our paper raises several avenues for further research. Firstly, none of the approaches considered for stabilising the weights helped address the issue of extreme weights of the IV-W method. Other ad-hoc methods such as identifying patients with extreme weights, removing them, and then re-estimating the weights may be of interest [30], but it is doubtful to be helpful with weak IV strength. Moreover, such methods often cause bias. Hence, more principled stabilisation methods for of IV-W are of interest before it can be used reliably outside of cases of a strong IV. Secondly, our simulation study assumed that outcome and treatment assignment models were correctly specified. In practice, this is unlikely to be the case due to non-linear relationships and interactions between the outcome, treatment, and confounders [31]. Assessing the robustness of IV-G and IV-W to model misspecification was beyond the scope of this paper but constitutes an interesting area for further research. Thirdly, our paper focused on binary time-varying IVs as the current implementation of IV-W requires a binary instrument to facilitate the calculation of the weights. However, many IVs in observational settings, such as the one considered in the case-study, are typically measured on the continuous scale. The IV-G approach can readily incorporate continuous IVs, but further work is required to assess the viability of using IV-W with continuous IV or treatment. Fourthly, our case-study exploited the potential of preference-based IVs in the context of rheumatoid arthritis and using registry data. Exploring this source of IV in other disease contexts and types of real-world data, such as electronic health records, would help establish its potential as a time-varying IV. There is also a question as to whether the adopted conditional F-test is always relevant guide of instrument strength in settings with time-varying IVs. Development of a bespoke test of IV strength over time is an interesting area of methodological development.

## Conclusions

In conclusion, the proposed IV-based g-estimation approach can be reliably used in the evaluation of time-varying treatments. The IV weighting approach offers an accessible alternative to g-estimation, but was found to work well only when the time-varying IV was strongly associated with treatment assignment at each time point. Our case-study suggested that this may not be the case in real-world applications using physicians preference as there may be limited change in the instrument over time, reducing the strength of the IV.

## Figures and Tables

**Fig. 4:**
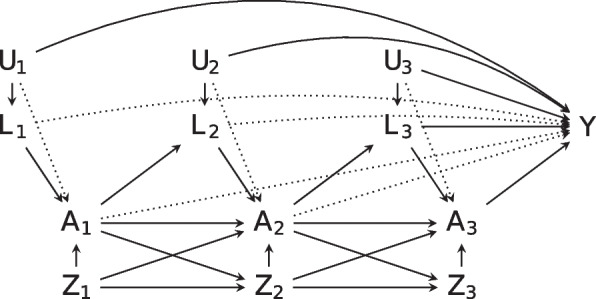
DAG displaying a more complex setup of time-varying data for $T=4$. Compared to Fig. 1, the complex scenario allows for direct associations between the treatments $A_t$, and for past values of the IV and treatment to affect the subsequent treatment and IV measurement respectively

**Fig. 5:**
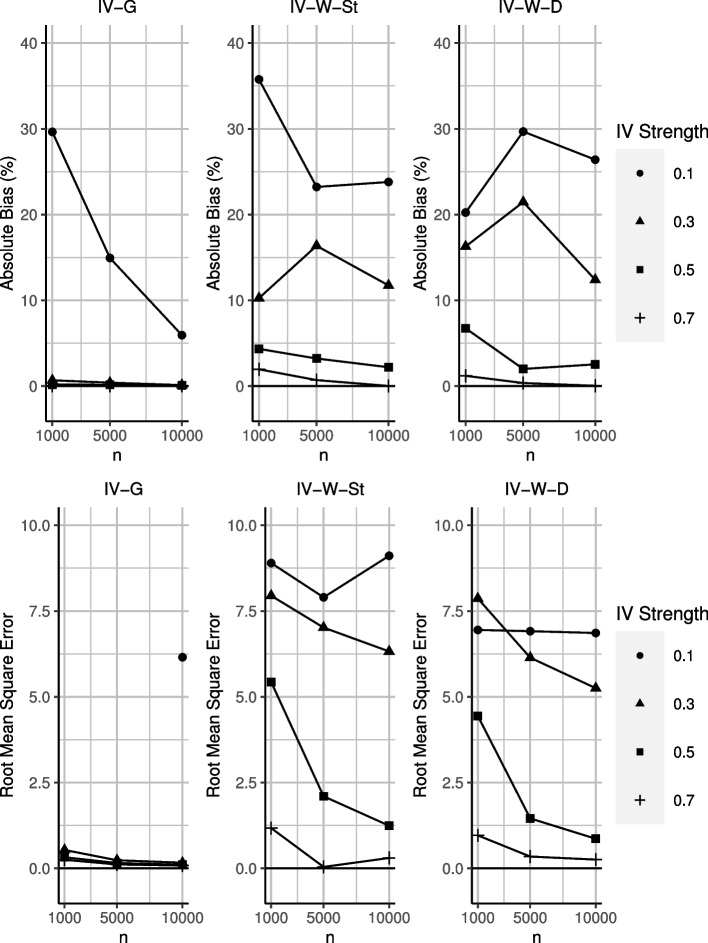
Absolute bias and root mean square error for complex simulation, based on 1000 bootstraps. Data points not shown far exceeded the upper bound of the y-axis

**Fig. 6:**
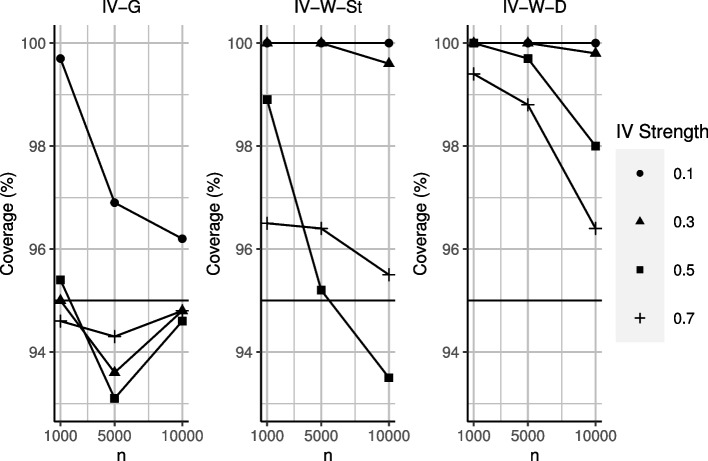
CI coverage results from IV-G and IV-W. IV-W results for scenarios with weak IV are not shown as they exceeded the upper bound of the y-axis. Nominal coverage is 95%

## Data Availability

The data that support the findings of this study are available from FORWARD, the National Data Databank for Rheumatic Diseases, but restrictions apply to the availability of these data, which were used under license for the current study, and so are not publicly available. Data are however available from the authors upon reasonable request and with permission of FORWARD.

## Appendix

### Complex simulations

In order to stress test the methods of the paper, we considered a more complicated data setup than in Fig. 1, described by the DAG below in Fig. 4. This data setup was intended to assess the performance of methods when there exists associations between $A_{t-1}, Z_{t-1}$ and $A_{t}, Z_{t}$, creating a more complex set of associations. Such associations may occur in realistic scenarios, especially with preference-based instruments.

#### Simulations

We simulated data according this DAG using the same methods, with two differences

- The time-varying instrument $Z_t$ is simulated as.
  $$\begin{aligned} logit(P(Z_t\!&=\!1|Z_{t-1},A_{t-1}))=\!\sigma _{0t}\!+\!\sigma _{1t}Z_{t-1}\!+\!\sigma _{2t}A_{t-1} \end{aligned}$$
  where we now set $\sigma _{0t}=\sigma _{2t}=0.5$, adding a dependence on $A_{t-1}$.
- $A_t$ is simulated as in [14] as
  $$\begin{aligned} & P(A_t|A_{t-1},Z_t,Z_{t-1},L_t,U_t)\\ =&pnorm(\mu _0+\mu _1 L_t+\mu _2 U_t+\mu _3 A_{t-1}+\mu _4 Z_{t-1})(1-\Delta _t)+Z_t\Delta _t. \end{aligned}$$
- In the complex setup we instead have $(\mu _0,\mu _1,\mu _2,\mu _3,\mu _4)=(-1,1,1,5,5)$ for all *t*. This creates much stronger associations between *U*, *L*, *Z* and *A*, as well as directly including an influence of $Z_{t-1}$ and $A_{t-1}$ on $A_t$. Scanrios with direct relations between $L_{t-1}$ and $L_t$ or $U_{t-1}$ and $U_t$ were considered but did not have a noticeable effect on results.

We performed the simulation analyses on this complex data in the exact same way as in the main paper. The results are displayed in Figs. 5 and 6 in Appendix and Tables 5 and 6 in Appendix. The results imply broadly the same conclusions as the setup in the main paper. Both g-estimation can handle datasets with more complex and stronger relationships. However, results broke down at higher samples sizes and IV strengths. For g-estimation this occurred due to the Z not being as jointly predictive, indicated by the conditional F statistics in Table 7 in Appendix. As before, bias occurred once these statistics reach or go below 10. For the inverse weighted procedure, the issue was likely due to further instability in the weighting terms.

**Table 5:** Complex simulation results for IV-G, based on b=1000 bootstrapped samples

*n*	*Cor*(*Z*)	Bias	RMSE	MCE	Coverage
10000	0.7	-0.002	0.084	0.003	94.8
10000	0.5	-0.004	0.105	0.003	94.6
10000	0.3	-0.006	0.164	0.005	94.8
10000	0.1	-0.297	6.15	0.19	96.2
5000	0.7	-0.002	0.114	0.004	94.3
5000	0.5	-0.008	0.155	0.005	93.1
5000	0.3	-0.020	0.237	0.007	93.6
5000	0.1	-0.747	28.740	0.909	96.9
1000	0.7	-0.002	0.251	0.008	94.6
1000	0.5	0.009	0.324	0.010	95.4
1000	0.3	-0.034	0.537	0.017	95.0
1000	0.1	1.482	57.576	1.821	99.7

**Table 6:** Complex simulation results for IV-W, based on b=1000 bootstrapped samples

Weight	*n*	*Cor*(*Z*)	Bias	MSE	MCE	Coverage
St	10000	0.7	0.001	0.303	0.010	95.7
St	10000	0.5	-0.110	1.243	0.041	96.7
St	10000	0.3	0.587	6.323	0.216	100.0
St	10000	0.1	1.190	9.111	0.316	100.0
Delta	10000	0.7	-0.002	0.256	0.008	96.3
Delta	10000	0.5	-0.127	0.863	0.027	96.0
Delta	10000	0.3	0.620	5.252	0.178	100.0
Delta	10000	0.1	1.320	6.861	0.231	100.0
St	5000	0.7	-0.035	0.042	0.013	96.1
St	5000	0.5	-0.161	2.100	0.070	98.7
St	5000	0.3	0.818	7.019	0.241	100.0
St	5000	0.1	1.168	7.900	0.269	100.0
Delta	5000	0.7	-0.018	0.344	0.011	96.8
Delta	5000	0.5	-0.100	1.456	0.048	97.2
Delta	5000	0.3	1.073	6.141	0.210	100.0
Delta	5000	0.1	1.484	6.913	0.232	100.0
St	1000	0.7	-0.098	1.174	0.038	96.3
St	1000	0.5	0.217	5.434	0.186	100.0
St	1000	0.3	0.513	7.948	0.272	100.0
St	1000	0.1	1.788	9.000	0.306	100.0
Delta	1000	0.7	-0.060	0.961	0.031	96.2
Delta	1000	0.5	0.337	4.444	0.153	99.7
Delta	1000	0.3	0.814	7.868	0.268	100.0
Delta	1000	0.1	1.012	6.940	0.235	100.0

**Table 7:** Average and Minimum Conditional F-statistics for each simulation setup over the 1000 bootstraps

Mean (Minimum) Conditional F-Statistic					
*n*	*Cor*(*Z*)	Sim	$\varvec{A}_{\varvec{1}}$	$\varvec{A}_{\varvec{2}}$	$\varvec{A}_{\varvec{3}}$
10000	0.7	Complex	6883 (5844)	7745 (6625)	11567 (9677)
10000	0.5	Complex	1815 (1679)	2103 (1679)	4019 (3084)
10000	0.3	Complex	224 (140)	252 (150)	673 (304)
10000	0.1	Complex	15.7 (1.4)	20.1 (1.5)	72.2 (1.9)
5000	0.7	Complex	3448 (2871)	3875 (3118)	5802 (4296)
5000	0.5	Complex	907 (695)	1052 (748)	2011 (1488)
5000	0.3	Complex	114 (53)	129 (57)	350 (114)
5000	0.1	Complex	8.6 (1.0)	12.3 (1.0)	51 (1.4)
1000	0.7	Complex	688 (416)	780 (435)	1166 (597)
1000	0.5	Complex	181 (89)	211 (96)	398 (145)
1000	0.3	Complex	24 (1.9)	28 (1.9)	82 (2.5)
1000	0.1	Complex	3.2 (0.9)	8.1 (1.0)	15.7 (1.2)
10000	0.7	Simple	8852 (7791)	7805 (6793)	7868 (6875)
10000	0.5	Simple	3199 (3730)	2815 (2364)	2838 (2437)
10000	0.3	Simple	1000 (784)	876 (689)	885 (655)
10000	0.1	Simple	118 (58)	105 (19)	106 (47)
5000	0.7	Simple	4435 (3743)	3901 (3216)	3923 (3198)
5000	0.5	Simple	1596 (1258)	1397 (1093)	1414 (1123)
5000	0.3	Simple	496 (334)	433 (290)	439 (288)
5000	0.1	Simple	55 (21)	49 (14)	49 (15)
1000	0.7	Simple	878 (603)	784 (487)	785 (466)
1000	0.5	Simple	311 (173)	276 (136)	277 (163)
1000	0.3	Simple	92 (44)	81 (33)	82 (28)
1000	0.1	Simple	7.3 (0.9)	6.9 (0.8)	6.8 (0.9)

Note that we denote ‘Simple’ as the simulation performed in the main body of the paper

**Table 8:** Standardised differences of key confounders between groups of doctors preference by prescription proportion at baseline

Covariate	Standardised difference
Dichotomised Age 1=>Mean	0.064
Smoke	0.069
Heart Disorder	0.006
Disease Activity Score $>3.2$	0.059
Medicare	0.136
Male	0.055
Total DMARD Count	0.055
White	0.003
0-10 Pain Scale	0.102
Diabetic	0.093
Infection	0.026
Duration of RA Symptoms in Years	0.019
0-9 Comorbidity Index	0.164
HAQ Disability Score	0.093
Cancer	0.006
Taking csDMARD	0.069
EQ-5D Score	0.107
Pulmonary Disorder	0.100
Any Opioid	0.049

*HAQ* Health Assessment questionnaire score, *Symp Years* Years with RA symptoms, *DAS* Disease Activity Score

### IV relevance and proof of relation between MSMs and SNMMs

#### Formal definition of IV relevance

We present the formal definition of IV relevance as given in [6]. Define

$$\begin{aligned} \delta _{ij}=E(A_i|Z_j=1)-E(A_i|Z_j=0) \end{aligned}$$

to represent the difference in expectation in $A_t$ under different levels of $Z_j$. We can write the full set of such comparisons where $i, j<T$ as a matrix *D*.

$$\begin{aligned} D = \left[ \begin{array}{cccc} \delta _{11}& \delta _{12} & \ldots & \delta _{1(T-1)}\\ \delta _{21} & \delta _{22} & \ldots & \delta _{2(T-1)}\\ \vdots & \vdots & \ddots & \vdots \\ \delta _{(T-1)1} & \delta _{(T-1)2} & \ldots & \delta _{(T-1)(T-1)} \end{array}\right] \end{aligned}$$

IV relevance requires that the instrument it each time *t* captures a change in the $A-Z$ relationship distinct from the other *Z*. For this we must have that *D* rank at least the number of treatment periods. In this case specifically, we must have that $rank(D)=T-1$.

To put another way, we cannot express the variation in *A* captured by $Z_t$ as some linear combination of the $Z_{-t}$. Note that this condition does not ensure that there is sufficient instrument strength to avoid weak instrument bias.

#### Relating the parameters of a SNMM to the ATE

Suppose that the three main IV assumptions hold, and that we have no effect modification of the effect of $A_t$ on Y by any past *A* or *Z*. For generalising purposes we suppose that $Z_t$ is conditionally exhangeable with *Y*(*a*) given past treatment and instrument history $M_t$. Recall the SNMM of the paper

$$\begin{aligned} E[Y(\bar{a}_t,0)-Y(\bar{a}_{t-1},0)|\bar{A}_t=\bar{a}_t, \bar{Z}_t]=\beta _t a_t. \end{aligned}$$

We show that under specific assumptions. The causal parameters $\beta _t$ can be related to those of the MSM

$$\begin{aligned} E[Y(\bar{a})]=\beta _0+\sum _{t=1}^T \beta _t a_t \end{aligned}$$

and thus the ATE can be estimated. We first show the relation for time $t=3$.

$$\begin{aligned} \beta _3 & =E[Y(1,1,1)-Y(1,1,0)|\bar{A_3}=(1,1,1), \bar{Z}_3]\\ & =E[Y(1,1,1)-Y(1,1,0)|\bar{A_3}=(1,1,0), \bar{Z}_3] \end{aligned}$$

By our assumption that the expectation is the same in the treated and untreated. Now by also invoking the no current treatment interaction assumption, this expectation is the same for any combination of $Z_3$ or $A_3$ We then have that this is equal to

$$\begin{aligned} =E[Y(1,1,1)-Y(1,1,0)|\bar{A_2}=(1,1), \bar{Z}_2] \end{aligned}$$

Repeating this process using the no current treatment interaction property at $t=2$, this is equal to, by the same reasonings

$$\begin{aligned} =E[Y(1,1,1)-Y(1,1,0)|A_1=1, Z_1] \end{aligned}$$

Now taking the SNMM at time $t=2$

$$\begin{aligned} \beta _2=E[Y(1,1,0)-Y(1,0,0)|\bar{A_2}=(1,1), \bar{Z}_2] \end{aligned}$$

By the same reasoning, using the no current treatment interaction at $t=2$, this is equal to

$$\begin{aligned} & =E[Y(1,1,0)-Y(1,0,0)|\bar{A_2}=(1,0), \bar{Z}_2]\\ & =E[Y(1,1,0)-Y(1,0,0)|A_1=1, Z_1] \end{aligned}$$

We therefore have

$$\begin{aligned} \beta _1+\beta _2 & = E[Y(1,1,0)-Y(1,0,0)|A_1=1, Z_1]\\ & \quad +E[Y(1,1,1)-Y(1,1,0)|A_1=1, Z_1]\\ & =E[Y(1,1,0)-Y(1,0,0)+Y(1,1,1)\\ & \quad -Y(1,1,0)|\bar{A_1}=(1), Z_1]\\ & =E[Y(1,1,1)-Y(1,0,0)|\bar{A_1}=(1), Z_1] \end{aligned}$$

Lastly The SNMM at $t=1$ states

$$\begin{aligned} \beta _1=E[Y(1,0,0)-Y(0,0,0)|A_1=1, Z_1] \end{aligned}$$

Taking its sum with the above equation yields

$$\begin{aligned} \beta _1+\beta _2+\beta _3 & =E[Y(1,1,1)-Y(1,0,0)+Y(1,0,0)\\ & \quad -Y(0,0,0)|A_1=1, Z_1]\\ & =E[Y(1,1,1)-Y(0,0,0)|A_1=1, Z_1] \end{aligned}$$

Finally, appealing to the no current treatment interaction at time t=1, we arrive at

$$\begin{aligned} E[Y(1,1,1)-Y(0,0,0)]=\beta _1+\beta _2+\beta _3 \end{aligned}$$

which is the ATE.

For completeness, suppose that conditional exhangeability of $Z_t$ also requires conditioning on some subset of variables $M_t$, which may include measured confounders $L_t$. The SNMM is then

$$\begin{aligned} E[Y(\bar{a}_t,0)-Y(\bar{a}_{t-1},0)|\bar{A}_t=\bar{a}_t, \bar{Z}_t,\bar{V}_t]=\beta _t a_t. \end{aligned}$$

We must assume that this expectation is the same in both the treated and untreated for all *t* (now including a conditioning on $M_t$. From here, the proof follows the same way).

## Abbreviations

EHR: Electronic Health Record
IV: Instrumental Variable
RA: Rheumatoid Arthritis
DMARD/bDMARD: (biologic)Disease Modifying Anti-rheumatic Drugs
EQ-5D: EuroQOL-5D score
QALY: Quality Adjusted Life Year
ATE: Average Treatment Effect
HAQ: Health Assessment Questionnaire
DAS: Disease Activity Score
